# Minimally invasive versus open right hepatectomy: comparative study with propensity score matching analysis

**DOI:** 10.1186/s12893-020-00919-0

**Published:** 2020-10-30

**Authors:** Vinícius Campos Duarte, Fabricio Ferreira Coelho, Alain Valverde, Divia Danoussou, Jaime Arthur Pirola Kruger, Kevin Zuber, Gilton Marques Fonseca, Vagner Birk Jeismann, Paulo Herman, Renato Micelli Lupinacci

**Affiliations:** 1 Liver Surgery Unit, Digestive Surgery Division, Department of Gastroenterology, University of São Paulo School of Medicine, Rua Dr. Enéas de Carvalho Aguiar, 255-9º Andar-sala 9025, São Paulo, SP CEP 05403-900 Brazil; 2 Digestive Surgery Unit, Diaconesses Croix Saint Simon Hospital, 125, Rue d’Avron, 75020 Paris, France; 3 Research and Biostatistics Unit, Rothschild Foundation Hospital, Paris, France; 4 AP-HP, Department of Digestive, Oncologic and Metabolic Surgery, Ambroise Paré Hospital, Boulogne-Billancourt, France; 5 Versailles St-Quentin-en-Yvelines/Paris Saclay University, UFR des Sciences de la Santé Simone Veil, Montigny-Le-Bretonneux, France

**Keywords:** Hepatectomy, Laparoscopy, Minimally invasive surgery, Hepatic neoplasms/surgery, Comparative study, Propensity score

## Abstract

**Background:**

Minimally invasive liver resections (MILRs) have been increasingly performed in recent years. However, the majority of MILRs are actually minor or limited resections of peripheral lesions. Due to the technical complexity major hepatectomies remain challenging for minimally invasive surgery. The aim of this study was to compare the short and long-term outcomes of patients undergoing minimally invasive right hepatectomies (MIRHs) with contemporary patients undergoing open right hepatectomies (ORHs)

**Methods:**

Consecutive patients submitted to anatomic right hepatectomies between January 2013 and December 2018 in two tertiary referral centers were studied. Study groups were compared on an intention-to-treat basis after propensity score matching (PSM). Overall survival (OS) analyses were performed for the entire cohort and specific etiologies subgroups

**Results:**

During study period 178 right hepatectomies were performed. After matching, 37 patients were included in MIRH group and 60 in ORH group. The groups were homogenous for all baseline characteristics. MIRHs had significant lower blood loss (400 ml vs. 500 ml, P = 0.01), lower rate of minor complications (13.5% vs. 35%, P = 0.03) and larger resection margins (10 mm vs. 5 mm, P = 0.03) when compared to ORHs. Additionally, a non-significant decrease in hospital stay (ORH 9 days vs. MIRH 7 days, P = 0.09) was observed. No differences regarding the use of Pringle’s maneuver, operative time, overall morbidity or perioperative mortality were observed. OS was similar between the groups (P = 0.13). Similarly, no difference in OS was found in subgroups of patients with primary liver tumors (P = 0.09) and liver metastasis (P = 0.80).

**Conclusions:**

MIRHs are feasible and safe in experienced hands. Minimally invasive approach was associated with less blood loss, a significant reduction in minor perioperative complications, and did not negatively affect long-term outcomes.

## Background

Minimally invasive liver resections (MILRs) have been increasingly performed in recent years. The available results have shown its safety, feasibility, and potential benefits over open liver resections (OLRs) [1, 2]. Observational studies and meta-analysis showed that laparoscopy decreased intraoperative bleeding, postoperative complications, and length of hospital stay when compared with OLRs [2, 3]. Recently, robotic-assisted surgery has been described as an alternative to laparoscopy for MILRs, with equivalent results for both short and long-term outcomes [4–6].

Although an impressive outspreading has been observed, most MILRs are actually minor or limited resections of peripheral lesions, mainly located in the anterolateral segments of the liver [3, 7, 8]. Due to the complexity of the operation and concerns about safety, major hepatectomies (resection of ≥ 3 contiguous segments) and notably anatomical right hepatectomies remain challenging for minimally invasive surgery [9–11].

Thus, the aim of this study was to compare the short and long-term outcomes of patients undergoing minimally invasive right hepatectomies (MIRHs) with contemporary patients undergoing conventional open right hepatectomies (ORHs) from two tertiary referral centers.

## Methods

Data of consecutive patients undergoing minimally invasive (laparoscopic and robotic) and open major hepatectomies between January 2013 and December 2018 were collected from prospective databases maintained by two tertiary referral centers. For the purpose of this study, only anatomical right hepatectomies were included. The Ethics Committee of both institutions approved this research protocol. The study was conducted following STROBE (Strengthening the Reporting of Observational studies in Epidemiology) recommendations [12].

The exclusion criteria were patients submitted to two-stage hepatectomy or ALPPS (Associating liver partition and portal vein ligation for staged hepatectomy); surgery for hilar cholangiocarcinoma; synchronous colorectal and liver resections, and patients with incomplete data.

The indication for the surgical procedure was carried out after discussion in a multidisciplinary meeting. Patients were evaluated for suitability of the minimally invasive approach according to tumor location, quality of the non-tumoral liver parenchyma, and clinical status. Selection criteria for MIRH were one or more lesions completely resectable with an anatomical right hepatectomy. Patient characteristics, such as body mass index (BMI) or previous abdominal surgery, were not contraindications for MIRH. The only contraindications to MIRH were invasion of the inferior vena cava, invasion of the main right portal vein, and need for vascular or biliary reconstruction. The benign or malignant nature of the tumor was not a contraindication.

Liver resections were defined according to Brisbane terminology [13]. ORH was defined as those performed through incisions as J-shape incision, “Chevron” or “Mercedes”. Succinctly, ORH was performed as follows: first, a classical extrahepatic hilar approach or “glissonian approach” was performed. After inflow control, the right hemiliver was fully mobilized including dissection of the hepatic parenchyma from the inferior vena cava after ligation of accessories hepatic veins; at the end of the mobilization right hepatic vein was encircled on a vessel loop. Finally, parenchyma transection was performed following the ischemic line using harmonic scalpel and bipolar forceps. Middle hepatic vein was preserved whenever it was possible without compromising surgical margins. Intermittent Pringle’s maneuver was used if needed in order to reduce operative blood loss.

Laparoscopic resections were performed using 5 or 6 ports located in right hypochondrium. Patients were positioned in a 30-degree reverse-Trendelenburg position and camera was positioned at right midclavicular line. The pneumoperitoneum pressure was set to 12 mmHg. Right pedicle control was performed by classical extrahepatic hilar dissection or extrahepatic “glissonian approach” as previously described [14]. Laparoscopic ultrasound was used to localize intrahepatic lesions and identify vascular structures. Parenchyma transection was performed using harmonic scalpel and bipolar forceps and right hepatic vein was sectioned inside the liver parenchyma using stapler. Surgical specimen was retrieved through a Pfannenstiel incision.

Robotic cases were performed using the da Vinci Si Surgical System from Intuitive Surgical Inc. (Sunnyvale, USA) with four robotic arms. Patients were positioned in a 30-degree reverse-Trendelenburg position. A 12-mm trocar was used to place a camera and three 8-mm ports were used for the instrument arms. Inflow vascular structures and the right biliary duct were dissected and then ligated with clips, *Hem-o-locks* or stapler. After complete right hemiliver mobilization, the liver parenchyma was divided with a combination of harmonic scalpel and the bipolar forceps. A “drop-in” ultrasound was used to localize intrahepatic lesions and identify vascular structures. Finally, right hepatic vein was sectioned inside the liver parenchyma using stapler.

The following preoperative characteristics were studied: age, gender, BMI, preoperative laboratory tests, presence of comorbidities, American Society of Anesthesiologists (ASA) physical status score, diagnosis, size and location of the lesions, previous abdominal surgeries, and presence of chronic liver disease. Perioperative variables studied were: operative time, estimated blood loss (EBL), pedicle clamping time, transfusion requirement, conversion rate, length of hospital stay, postoperative complications, rehospitalization and mortality.

Postoperative morbidity was defined as complications occurring during the first 90 postoperative days and was stratified according to the Dindo-Clavien classification [15]. Postoperative biliary fistula was defined following the criteria proposed by the International Study Group of Liver Surgery [16]. Postoperative mortality was defined as death within 90 days after liver resection.

On final pathology report, both the rate of radical resections (R0) and the width of tumor-free margin (mm) were analyzed. Resections were defined as R0 when microscopically margins were ≥ 1 mm and R1 when margins were < 1 mm. Overall survival (OS) was defined as the time interval between the date of liver resection and the date of death or the most recent date of follow-up if the patient was alive.

Primary outcome was overall morbidity rate. Additional outcomes considered to be of interest and help interpret the results were chosen as secondary outcomes and included: operative time, EBL, blood transfusion rate, free surgical margins, hospital stay, rehospitalization, and mortality.

### Statistical analysis

Continuous data were expressed as median and interquartile range or mean and standard deviation. Comparisons were performed using Mann–Whitney U test or T test. Categorical variables were expressed as percentage and compared using Fisher’s exact test or Chi-squared test. A P value < 0.05 was considered statistically significant. OS was assessed using the Kaplan–Meier method and curves were compared with the log-rank test. Survival analyses were performed for the entire cohort and specific etiologies subgroups (malignant liver tumors and metastasis).

**Fig. 1:**
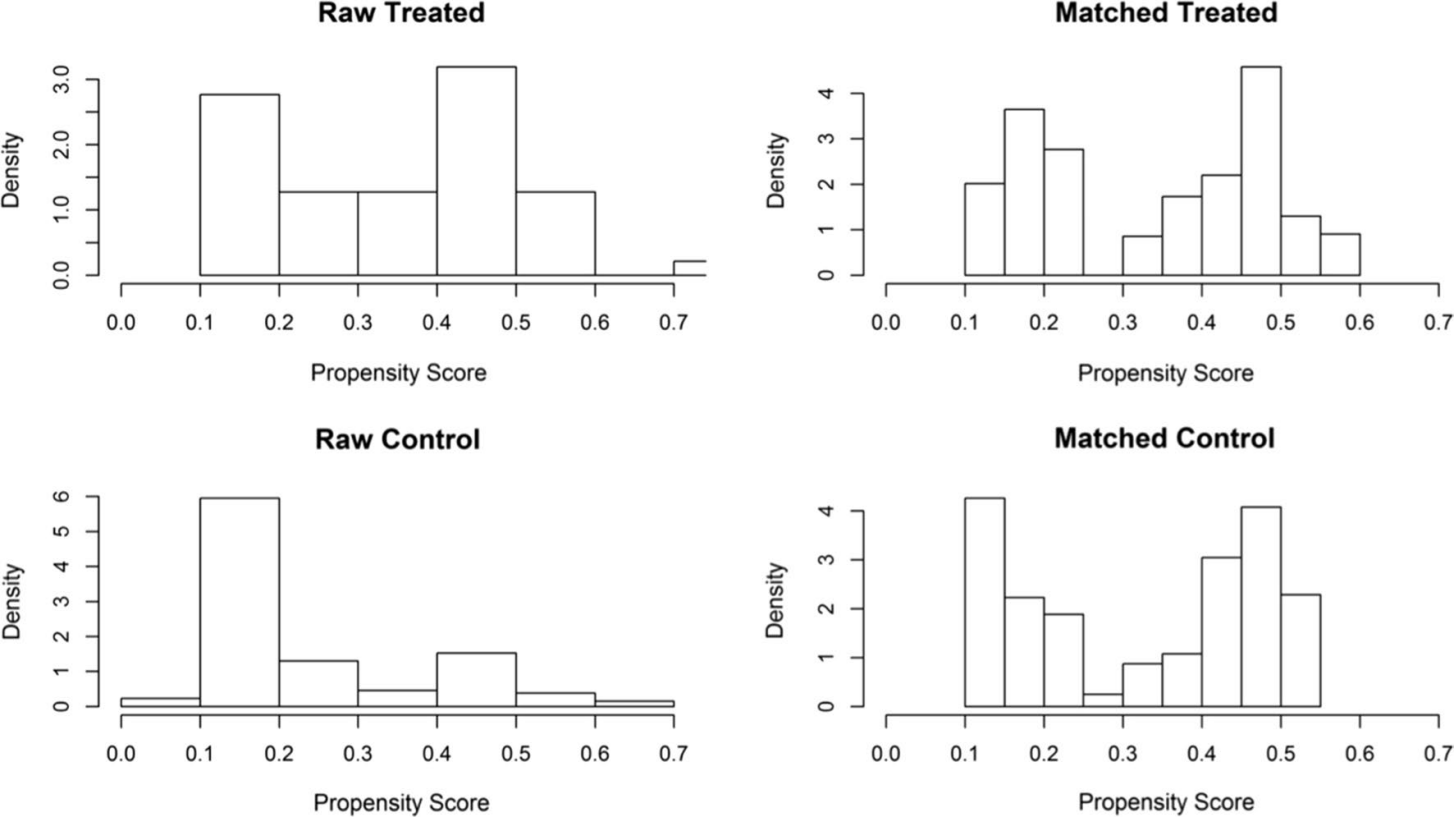
Density histograms for open (control) and laparoscopic (treated) groups before and after propensity score matching using the model with the variables: ASA score, number of nodules, previous abdominal surgery and diagnosis

Propensity score matching (PSM) was used to control possible confounding bias [17]. The PSM was constructed using age, ASA score, number of nodules, previous abdominal surgery and diagnosis. The nearest neighbor method was used with a caliper of 0.20. The histograms after adjustment (matched) are very similar while the histograms before adjustment (raw) were different (Fig. 1). Comparisons were performed on an intention-to-treat basis; therefore, converted procedures were maintained in the MIRH group.

## Results

During the study period 178 right hepatectomies were performed in both centers, 47 MIRH (including 16 robot-assisted and 31 laparoscopic resections) and 131 ORH. After match by PSM, 37 patients were included in MIRH group and 60 in ORH group. Clinical characteristics of each group before and after matching are shown on Table 1.

**Table 1:** Clinical preoperative characteristics before and after matching with propensity score matching (PSM)

Variable	ORH N = 131	MIRH N = 47	P	ORH after PSM N = 60	MIRH after PSM N = 37		P	
Gender (%)			0.97				1	
Female	59 (45%)	22 (46.8%)		27 (45%)	17 (45.9%)			
Male	72 (55%)	25 (53.2%)		33 (55%)	20 (54.1%)			
Age (years)	58 [45–67]	59 [47.5–67]	0.88	59 [52.5–68.25]	59 [52–67]		0.80	
BMI (kg/m^2^)	25.3 [22.3–28.5]	25.4 [22.5–27.3]	0.80	25.2 [22.1–27.7]	25.6 [22.5–27.1]		0.84	
Obesity (%)	20 (17.4%)	5 (11.9%)	0.56	7 (13%)	3 (9.4%)		0.74	
ASA (%)			0.67				0.84	
1–2	108 (82.4%)	37 (78.7%)		48 (80%)	31 (83.8%)			
3	23 (17.6%)	10 (21.3%)		12 (20%)	6 (16.2%)			
Cardiologic comorbities (%)	13 (9.9%)	3 (6.4%)	0.57	7 (11.7%)	2 (5.4%)		0.48	
Pulmonary comorbities (%)	4 (3.1%)	2 (4.3%)	0.66	2 (3.3%)	2 (5.4%)		0.63	
Cirrhosis (%)	11 (8.4%)	11 (23.4%)	*0.02*	7 (11.7%)	7 (18.9%)		0.49	
Diagnostic (%)			0.06				0.87	
Benign	17 (13%)	9 (19.1%)		9 (15%)	7 (18.9%)			
Metastasis	87 (66.4%)	23 (48.9%)		33 (55%)	19 (51.4%)			
Malignant	26 (19.8%)	15 (31.9%)		18 (30%)	11 (29.7%)			
Nodules (%)			*0.03*				0.95	
1	41 (35.7%)	7 (15.9%)		13 (21.7%)	7 (18.9%)			
2–3	52 (45.2%)	23 (52.3%)		33 (55%)	21 (56.8%)			
≥ 4	22 (19.1%)	14 (31.8%)		14 (23.3%)	9 (24.3%)			
Previous abdominal surgery (%)	100 (76.3%)	20 (42.6%)	*< 0.0001*	36 (60%)	18 (48.6%)		0.38	
Chemotherapy (%)	77 (59.2%)	20 (42.6%)	0.07	28 (46.7%)	16 (43.2%)		0.91	
PVE (%)	24 (18.3%)	11 (23.4%)	0.59	12 (20%)	9 (24.3%)		0.80	

Values with statistical significance (P < 0.05)

*ORH* open right hepatectomy, *MIRH* minimally invasive right hepatectomy, *PSM* propensity score matching, *BMI* body mass index, *ASA* American Society of Anesthesiologists physical status score, *PVE* portal vein embolization

Before matching significant differences between groups were observed, including higher frequency of cirrhosis in MIRH group (23.4% vs. 8.4%, P = 0.02), multiple nodules resected in MIRH group (78.7% vs. 56.5%, P = 0.03), and more patients submitted to previous abdominal surgery in ORH group (76.3% vs. 42.6%, P < 0.0001). After matching, the groups became homogenous for all baseline characteristics (Table 1).

Intraoperative results are shown on Table 2. The conversion rate in the whole MIRH group was 4.2% (2 out of 47). After matching, conversion to open procedure was necessary in 5.4% (2 out of 37) of the cases, both due to technical difficulties and intraoperative bleeding. No difference regarding the use of Pringle’s maneuver, clamping time and operative time was observed. Significant higher blood loss (500 ml vs. 400 ml, P = 0.01) was observed in patients submitted to ORHs, despite no difference in blood transfusion rate was observed.

**Table 2:** Intraoperative outcomes before and after matching with propensity score matching (PSM)

Variable	ORH N = 131	MIRH N = 47	P	ORH after PSM N = 60	MIRH after PSM N = 37	P
Associated radiofrequency (%)	6 (4.6%)	1 (2.1%)	0.68	1 (1.7%)	1 (2.7%)	1
Pringle’s maneuver (%)	36 (27.5%)	8 (17%)	0.17	17 (28.3%)	6 (16.2%)	0.26
Clamping time (min) Median (interquartile range)	15 [14.5–30]	15 [6.25–40]	0.71	12 [0–30]	0 [0–7.5]	0.07
Operative time (min) Median (interquartile range)	390 [320–480]	360 [275–480]	0.43	390 [322.5–480]	360 [260–491.25]	0.42
EBL (ml) Median (interquartile range)	500 [400–1000]	475 [250–675]	*0.007*	500 [400–1000]	400 [215–600]	*0.01*
Transfusion (%)	37 (28.2%)	9 (19.1%)	0.25	17 (28.3%)	7 (18.9%)	0.42

Values with statistical significance (P < 0.05)

*ORH* open right hepatectomy, *MIRH* minimally invasive right hepatectomy, *EBL* estimated blood loss

We observed a 2 days reduction in hospital stay in the MIRH group, although it did not reach statistical significance (ORH 9 days vs. MIRH 7 days, P = 0.09). No significant difference was observed in overall morbidity between the groups (ORH 53.3% vs. MIRH 35.1%, P = 0.09); however, when stratified according to the Dindo-Clavien classification a lower rate of minor complications was observed in MIRH group (13.5% vs. 35%, P = 0.03). No differences were found in liver-related complications, major complications and perioperative mortality (Table 3).

**Table 3:** Postoperative results before and after propensity score matching (PSM)

Variable	ORH N = 131	MIRH N = 47	P	ORH after PSM N = 60	MIRH after PSM N = 37	P
Hospital stay (days) Median (interquartile range)	9 [7–15]	7 [6–12.75]	0.15	9 [6–14.25]	7 [6–11]	0.09
Reoperation (%)	4 (3.1%)	0 (0%)	0.57	3 (5%)	0 (0%)	0.28
Rehospitalization (%)	15 (11.5%)	4 (8.5%)	0.78	4 (7.7%)	4 (11.8%)	0.71
Overall morbidity (%)	71 (54.2%)	20 (42.6%)	0.17	32 (53.3%)	13 (35.1%)	0.09
Dindo-Clavien (%)						
I–II	44 (33.6%)	9 (19.1%)	0.07	21 (35%)	5 (13.5%)	*0.03*
III–IV	13 (9.9%)	11 (23.4%)	*0.03*	5 (8.3%)	8 (21.6%)	0.07
Perioperative mortality (%)	14 (10.7%)	0 (0%)	*0.02*	6 (10%)	0 (0%)	0.08
Liver-related complications						
Hemorrhage (%)	4 (3.1%)	1 (2.1%)	1	1 (1.7%)	1 (2.7%)	1
Biliary fistula (%)	17 (13%)	5 (10.6%)	0.80	8 (13.3%)	5 (13.5%)	1
Ascites (%)	16 (12.2%)	4 (8.5%)	0.60	9 (15%)	2 (5.4%)	0.20
Encephalopathy (%)	4 (3.1%)	1 (2.1%)	1	3 (5%)	0 (0%)	0.28
Size of largest lesion (mm) Median (interquartile range)	41.5 [22–80]	51.5 [25.75–75.75]	0.35	50 [22–102.5]	55 [25–78]	0.69
Surgical margins (%) Free	115 (87.8%)	40 (85.1%)	0.62	55 (91.7%)	34 (91.9%)	1
Margin width (mm) Median (interquartile range)	5 [1–9.5]	15 [5–77.5]	*0.0002*	5 [2–9.5]	10 [5–25]	*0.03*

Values with statistical significance (P < 0.05)

*ORH* open right hepatectomy, *MIRH* minimally invasive right hepatectomy

**Fig. 2:**
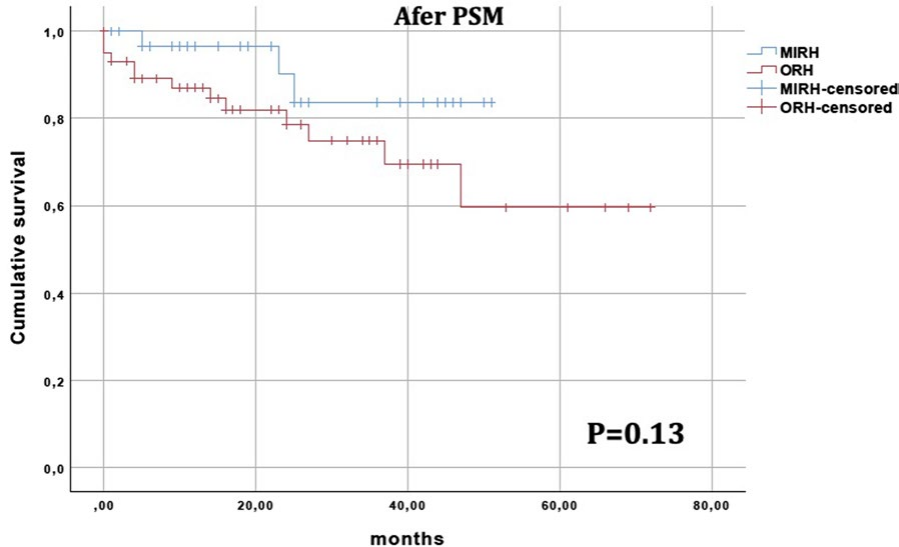
Kaplan–Meier overall survival curves after propensity score matching (PSM) for patients undergoing open right hepatectomies (ORH) and minimally invasive right hepatectomies (MIRH)

No difference on the clearance of surgical margins between groups was observed, but MIRHs showed larger resection margins (10 mm vs. 5 mm, P = 0.03). Survival analysis of the entire cohort after matching was similar between patients that underwent ORHs and MIRHs (P = 0.13, Fig. 2).

### Subgroup analysis

**Fig. 3:**
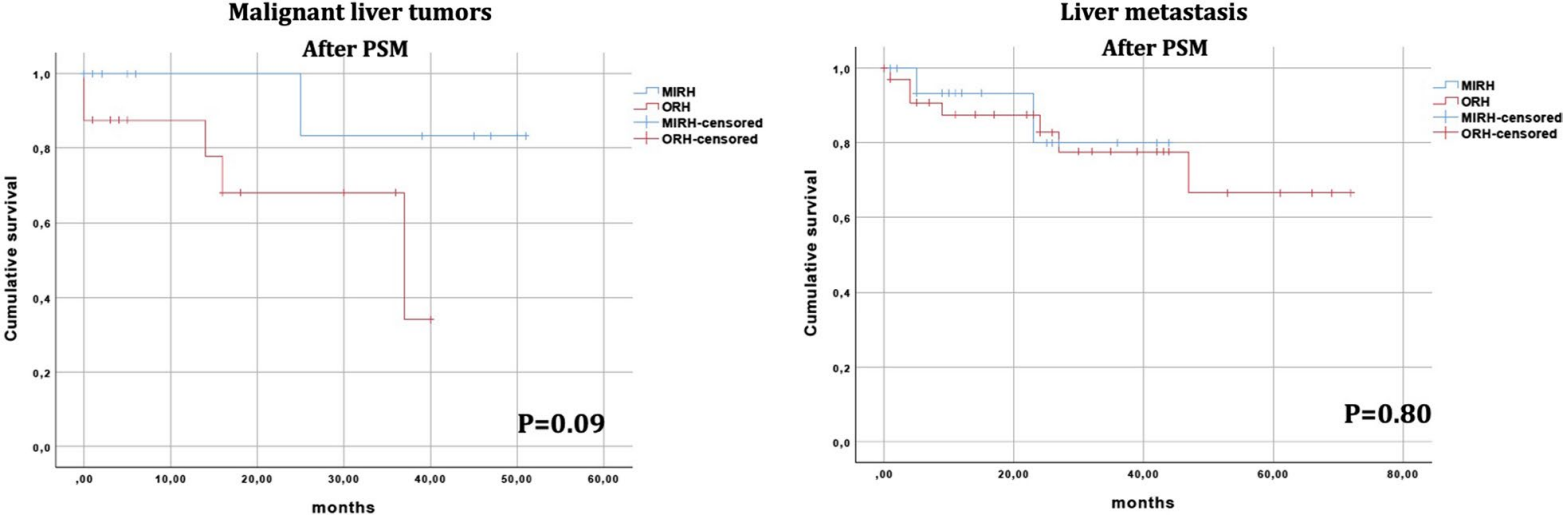
Overall survival curves for subgroups of patients with primary liver tumors and liver metastasis after propensity score matching (PSM). *ORH* open right hepatectomy, *MIRH* minimally invasive right hepatectomy

Survival analysis was performed in 2 subgroups: patients with malignant liver tumors and patients with liver metastasis. In both subgroups no significant difference in OS was observed after matching (Fig. 3).

## Discussion

The first MILRs reported at the beginning of the 1990s were basically wedge resections of peripheral lesions [18]. Subsequently, anatomic resections such as left lateral sectionectomy were performed [19]. The first major laparoscopic hepatectomy series was reported in 1997 by Hüscher et al. [20] using hybrid procedures for right-sided resections.

Although it has several theoretical advantages, only a small percentage of liver resections are actually performed by minimally invasive surgery. A recent French national database study showed that 15% of liver resections were performed through minimally invasive approach [21]. Similarly, Kim et al. [22] showed that less than 10% of all liver resections for benign lesions in the United States were minimally invasive.

Currently, minor laparoscopic resections in anterolateral segments and left lateral sectionectomy are considered the gold standard approach in many specialized centers [3, 8, 23]. However, resections of bilateral lesions, nodules in posterosuperior segments or in central locations of the liver (segments 1, 4a, 7, and 8), and major hepatectomies are still challenging [24, 25]. In fact, technical demands have limited major hepatectomies to highly-skilled surgeons in referral centers [10, 26]. Concerns during anatomical right liver resections are related to liver mobilization from the inferior vena cava, inflow and outflow control, and a large parenchymal transection area. Moreover, the learning curve for MILR can reach 45–75 procedures [11, 27].

Technical limitations of laparoscopic major resections were depicted in a recent survey including 27 specialized centers. While minimally invasive approach was used in 61.8% of left lateral sectionectomies, this percentage decreased to 24.8% for major hepatectomies [28].

Robot-assisted surgery has been increasingly employed as an alternative to laparoscopy for MILR, mainly in complex and major liver resections [29, 30]. Despite potential advantages, most of the available evidence demonstrated similar results between laparoscopic and robotic liver resections [5, 31, 32].

Few studies were addressed to study the results of minimally invasive major hepatectomies [10, 26, 33]. Only recently observational studies with high methodological quality have been published comparing open and minimally invasive major resections [34, 35]. Takahara et al. [36] using PSM showed advantages in terms of blood loss, length of hospital stay, and complications with the laparoscopic approach.

We observed a conversion rate of 5.4%, lower than observed in other series raging from 9 to 42%. Cipriani et al. [37] showed an 11% conversion rate in a European multicenter study. Similarly, Kasai et al. [33] observed a 17.7% conversion rate in a recent meta-analysis of individual data.

MILR is frequently associated with a longer operative time [34, 35]. However, we did not find any significant difference in operative time for patients undergoing MIRH. This finding can be explained by the increased experience with MILR, showing that the learning curve was overcome and surgical steps have been standardized to entail a significant reduction in operative time [38, 39]. In fact, were included in our study patients that underwent hepatectomy between 2013 and 2018, after overcome the learning curve with MILRs. Minimally invasive liver surgery program started at the University of São Paulo in 2005 and at the Diaconesses Croix Saint Simon Hospital in 2010. The 2 centers altogether have performed more than 550 minimally liver resections.

In accordance with previous studies [34, 36], we observed a significantly lower blood loss in the MIRH group. Factors that may have influenced this reduction are the development of new energy devices for liver transection, the image magnification afforded by laparoscopy, the pneumoperitoneum, and the widespread use of linear staplers for controlling hepatic pedicles and large vessels [1].

Several authors found a reduction in perioperative complications in the MILR group [36, 37]. However, in our study the overall morbidity (our primary end-point) presented a non-significant decrease (35.1% vs. 53.3%, P = 0.09), probably related to the small sample size of our casuistry. Although non statistically significant, this finding appears to be clinically relevant. Our data showed a decrease in Dindo-Clavien I–II complications (13.5% vs. 35%, P = 0.03), in accordance with the findings of a recent meta-analysis [33].

The reduction of hospital stay is an outcome frequently attributed to minimally invasive surgery [2, 36]. We observed a 2-day reduction in the MIRH group. Although not statistically significant, we considered this a consistent clinical benefit for patients subjected to major liver resections.

Concerning the oncological outcomes, there was no increase in R1 resections in MIRH group. This data is in accordance with other studies that found similar R0 resections, and wider margins associated with MILRs when compared to OLRs [2, 40].

Few comparative studies assessed the long-term results of minimally invasive resections [33, 41]. The fear of inferior oncological results in patients undergoing MILRs was not demonstrated by the available studies (33, 35). Similarly, in our study the OS rate in MIRH group was not inferior when compared to isolated ORH group. The same finding was observed in subgroups of patients operated for primary liver tumor and liver metastases.

Our study has the classical drawbacks of any retrospective analyses. For this reason, we focused only in right anatomical liver resections. Moreover, in order to minimize selection bias we excluded complex or associated procedures such as two-stage hepatectomies, hilar cholangiocarcinoma and synchronous resections. Finally, we used the PSM and observed that after matching, both groups were homogeneous in the main clinical and surgical characteristics. PSM was used to limit observed confounding factors, however it is important to state that PSM does not allow us to control for possible selection bias. Also, by introducing secondary outcomes and performing other comparisons, we could have increased the alfa risk and decay our results. Altogether, these points may limit the extent to which we can generalize our findings.

## Conclusion

In this bicentric study, MIRHs performed by experienced surgeons were feasible and safe. Compared with matched patients submitted to ORH, minimally invasive approach was associated with less blood loss, a significant reduction in minor perioperative complications and did not negatively affect long-term outcomes.

## Data Availability

The datasets used and/or analysed during the current study are available from the corresponding author on reasonable request.

## Abbreviations

MILRs: Minimally invasive liver resections
OLRs: Open liver resections
MIRHs: Minimally invasive right hepatectomies
ORHs: Open right hepatectomies
PSM: Propensity score matching
OS: Overall survival
STROBE: Strengthening the Reporting of Observational studies in Epidemiology
ALPPS: Associating liver partition and portal vein ligation for staged hepatectomy
BMI: Body mass index
ASA: American Society of Anesthesiologists
EBL: Estimated blood loss
